# siRNA-mediated inhibition of endogenous brain-derived neurotrophic factor gene modulates the biological behavior of HeLa cells

**DOI:** 10.3892/or.2022.8438

**Published:** 2022-11-02

**Authors:** Chun-Yan Sun, Zhang-Bo Chu, Jing Huang, Lei Chen, Jian Xu, Ao-Shuang Xu, Jun-Ying Li, Yu Hu

Oncol Rep 37: 2751–2760, 2017; DOI: 10.3892/or.2017.5569

Subsequently to the publication of the above article and a Corrigendum that was published to indicate corrections made to Fig. 7 (DOI: 10.3892/or.2021.7922; published online on January 5, 2021), a concerned reader drew the Editor's attention to the fact that, comparing between a pair of panels in the Figure, there was an overlapping section of data; moreover, this overlapping section contained apparent anomalies that could not be easily accounted for through a straightforward re-use of one of the data panels. The authors conceded that there was partial duplication between the images shown in Fig. 7B and F, although they were unable to access the related raw data as the experiments had been performed almost 10 years ago. Secondly, the authors informed the Editor that the corresponding author did not know he was on the author list at the time of submission.

Although the authors' were granted permission to publish the Corrigendum, the Editor now considers that the paper should be retracted on account of the uncertainties in the presented revised data and the authors' admission concerning the corresponding author. Therefore, this paper has been retracted from the Journal. The authors are in agreement with the decision to retract the article. The Editor apologizes for any inconvenience caused.

